# Human spinal cord organoids: A powerful tool to redefine gray matter and lower motor neuron pathophysiology in spinal cord injury

**DOI:** 10.4103/NRR.NRR-D-25-00111

**Published:** 2025-04-29

**Authors:** Maria Jose Quezada, Colin K. Franz

**Affiliations:** Department of Biomedical Engineering, Northwestern University, Evanston, IL, USA; Department of Physical Therapy and Human Movement Sciences, Northwestern University, Chicago, IL, USA; Biologics Laboratory, Shirley Ryan AbilityLab, Chicago, IL, USA; Physical Medicine and Rehabilitation, Northwestern University Feinberg School of Medicine, Chicago, IL, USA; Ken & Ruth Davee Department of Neurology, Northwestern University Feinberg School of Medicine, Chicago, IL, USA; Querrey Simpson Institute for Bioelectronics, Northwestern University, Technological Institute, Evanston, IL, USA

Human spinal cord organoids (hSCOs) offer a promising platform to study neurotrauma by addressing many limitations of traditional research models. These organoids provide access to human-specific physiological and genetic mechanisms and can be derived from an individual’s somatic cells (e.g., blood or skin). This enables patient-specific paradigms for precision neurotrauma research, particularly relevant to the over 300,000 people in the United States living with chronic effects of spinal cord injury (SCI).

In SCI, damage to both gray and white matter occurs, yet research has predominantly focused on white matter injury, especially the disruption of long axonal tracts. Achieving long-distance axon regeneration in the central nervous system remains a significant challenge. Meanwhile, the functional consequences of gray matter damage, such as disruptions to cells and circuits that regulate breathing, locomotion, and critical reflex pathways, are often underappreciated (Debenham et al., 2024). Advances in hSCO technology, including region-specific patterning based on developmental biology principles, offer new opportunities to study local gray matter damage. These models are complementary to traditional preclinical animal studies, which have provided foundational insights into SCI pathophysiology and therapeutic development (Zhang et al., 2014).

**Clinical implications of spinal cord injury gray matter pathology:** Efforts to improve sensorimotor recovery in SCI have traditionally emphasized damage to upper motor neuron axon tracts in the spinal cord. However, advances in clinical electrodiagnostic assessments have enabled a more precise distinction between motor deficits caused by upper motor neuron and lower motor neuron (LMN) dysfunction (Debenham et al., 2024). This distinction is critical for tailoring rehabilitation strategies and understanding injury progression, as LMN damage introduces unique challenges that significantly impact the prognosis for functional recovery and quality of life. Gray matter pathology, which affects LMNs and their associated circuits, plays a central role in many of these challenges. The following examples illustrate the diverse and significant impacts of gray matter pathology on function after SCI:

***Respiratory impairments (cervico-thoracic injuries)***: Damage to motor neurons, such as the phrenic motor pools at C3–C5, can cause severe respiratory complications, including atelectasis, pneumonia, respiratory failure, and chronic dependence on invasive mechanical ventilation. Injuries above C8 impair abdominal and intercostal muscles, weakening inspiration, cough, and secretion clearance. Specific deficits include loss of intercostal function (T1–T5) and progressive abdominal dysfunction (T5–T12). Preclinical models show damage to white matter and lateralized gray matter, including ventral horn neurons and interneurons.

***Upper limb recovery (cervical injuries)***: Differentiating upper motor neuron and LMN contributions to weakness is crucial for recovery strategies. Cervical SCI often impairs elbow extension (C7), hand opening (C8), and hand closing (C8). Nerve transfer surgery, which coapts a healthy donor nerve to a paralyzed muscle’s nerve, can restore critical functions if performed in well-selected patients. Timely assessment of LMN health is essential, as muscle denervation and fibrosis become irreversible within 12–18 months post-injury (Debenham et al., 2024). Early electrophysiological evaluations guide the timing and type of interventions offered and subsequent rehabilitation.

***Central pattern generators and reflex circuits (lower thoracic and lumbar injuries)***: The spinal cord acts as a “command center,” with central pattern generators (CPGs) generating rhythmic movements independent of brain input. Damage to CPGs and reflex circuits disrupts locomotion and other spinally regulated functions. Activity-dependent plasticity, such as through intensive gait training, can reorganize spinal locomotor circuits below the lesion, though outcomes vary and interactions with gray matter damage remain unclear (Guertin, 2014). While extensively studied in preclinical models, direct assessments of human CPG function in clinical practice remain an unmet need, limiting the ability to precisely target these circuits for rehabilitation.

***Autonomic dysfunctions (lumbo-sacral injuries)***: Gray matter damage in the sacral cord disrupts bladder, bowel, and sexual function. LMN injury causes areflexic bladder and bowel, urinary retention, incontinence, and heightened infection risks. Sexual dysfunction includes impaired erections and orgasm due to damaged sacral circuits. Sacral gray matter injury also affects pelvic floor motor neurons, compromising sphincter control and stability during voiding. Additionally, preganglionic neuron damage impairs vasodilation and pelvic organ blood flow, exacerbating erectile dysfunction. Loss of sensory input further disrupts reflexes and body awareness. Notably, surveys of individuals with SCI consistently rank restoration of bladder, bowel, and sexual function as among the most important recovery priorities, underscoring the urgency to address these deficits and enhance quality of life.

**Figure 1:**
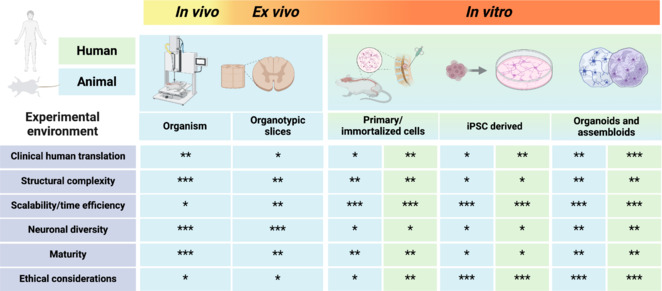
Comparative features of experimental models for spinal cord injury research. This figure summarizes the advantages and limitations of *in vivo*, *ex vivo*, and *in vitro* experimental models for spinal cord research. *In vivo* models (organisms) provide high structural complexity and neuronal diversity but have limited scalability and ethical concerns due to animal use. *Ex vivo* organotypic slices balance complexity and scalability but lack maturity. *In vitro* models, including primary/immortalized cells, induced pluripotent stem cells (iPSC)-derived systems, and organoids/assembloids, enable high scalability and good ethical viability. Organoids and assembloids stand out for their combination of translational relevance, neuronal diversity, and advanced engineering capabilities, while maintaining scalability and time efficiency. Created with BioRender.com. *: Low; **: medium; ***: high.

**Bridging gaps in traditional spinal cord injury models:** Multi-species *in vivo* models have provided valuable insights into the cellular, molecular, and pathophysiological responses to neurotrauma across acute and chronic stages. Compression and contusion animal models have been used to study neuropathological responses including inflammation and glial scarring, while transection models investigate axonal regeneration in white matter (Sharif-Alhoseini et al., 2017). Animal organotypic slice models, which maintain some of the tissue architecture, offer a platform to test independent factors in a single animal, reducing the number of samples required. Although animal models have been instrumental in exploring time-dependent cell death at varied distances from the lesion center, scientific and ethical considerations have compelled scientists to consider alternative preclinical models (**Figure 1**).

Building on these animal models, two-dimensional *in vitro* approaches provide higher throughput and time-efficient platforms to enhance experimental control. Cell types used for these models can be derived from animals or humans, including immortalized cell lines and primary cell cultures obtained from dissociated animal neural tissues (i.e., neurons, oligodendrocytes, astrocytes, and microglia) at early embryonic or postnatal stages. While embryonic stem cells are considered the gold standard due to inherent pluripotency and stable differentiation potential, the rising use of induced pluripotent stem cells (iPSC) offsets the ethical constraints to study human tissue of the central nervous system. iPSCs are patient somatic cells that are genetically reprogrammed to embryonic-like states with the capability to differentiate into multiple cell types. These provide a platform to develop personalized disease modeling and study genetic and neurotrauma interactions. These cells can self-assemble and differentiate into organ-specific cells, forming three-dimensional cultures called organoids, mimicking tissue structure and organ function. However, determining the cellular origin of iPSCs is important, as epigenetic memory and transcriptional profiles of donor cells can influence differentiation efficiency, regional identity, and functional maturation. Pluripotency assays and genome integrity tests are standard quality control measures conducted prior to organoid generation (Pașca et al., 2025). Over the last decade, human iPSC technology has advanced rapidly including more complex 3D cultures such as hSCO.

**Figure 2:**
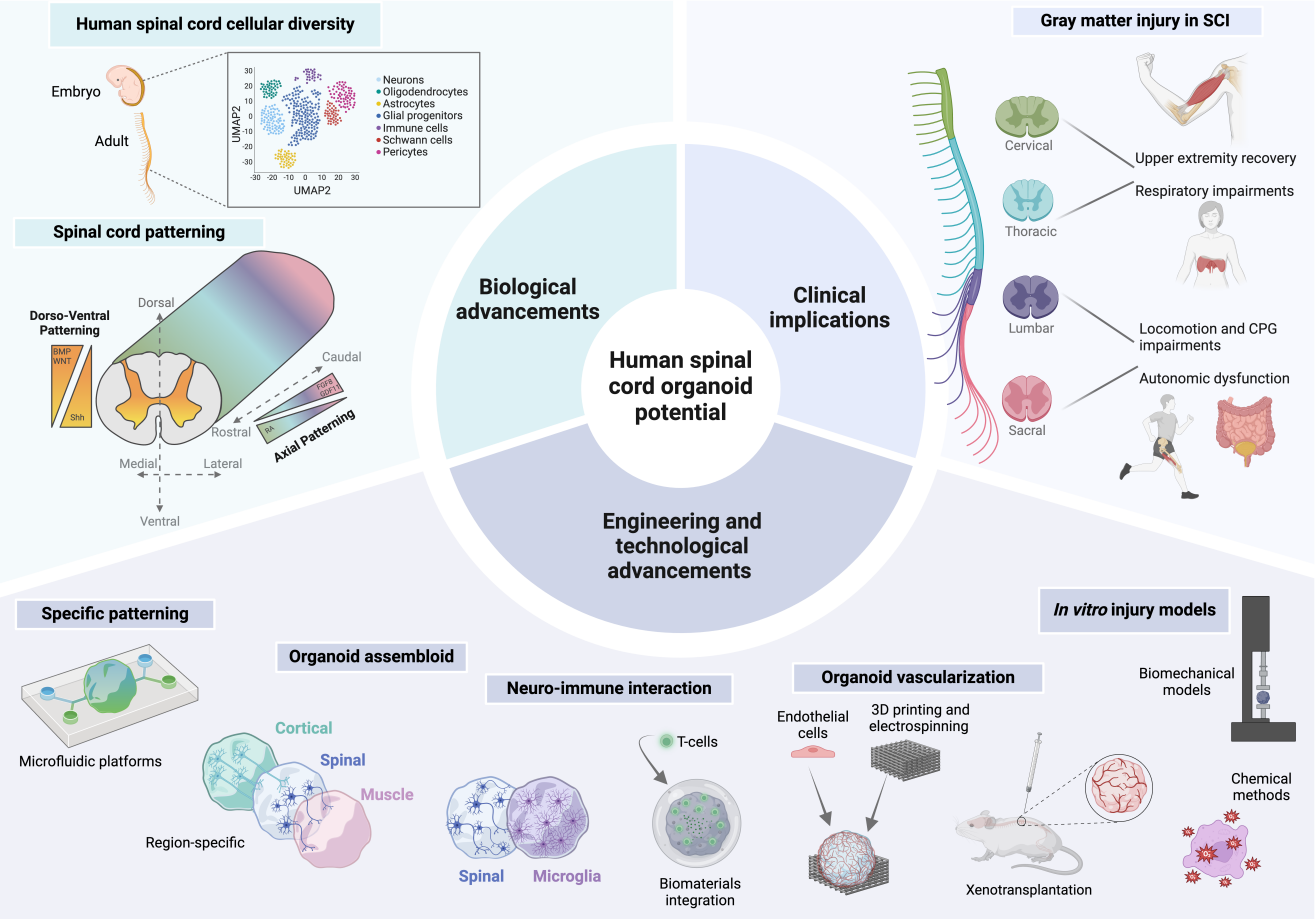
Advancing human spinal cord organoid research-potential and applications. This figure highlights the multifaceted potential of hSCOs in understanding SCI pathophysiology and advancing therapeutic strategies. Biological advancements include human spinal cord cellular diversity and precise spinal cord patterning through dorsoventral and axial gradients. Clinical implications focus on gray matter injury across spinal levels, impacting respiratory, upper limb, locomotor, and autonomic functions. Engineering and technological innovations encompass in vitro injury models, organoid vascularization via biomaterials and 3D printing, and neuro-immune interactions. Region-specific patterning and assembloids further enhance hSCO complexity, enabling comprehensive modeling of SCI mechanisms and recovery pathways. Created with BioRender.com. 3D: Three-dimensional; CPG: central pattern generator; hSCOs: human spinal cord organoids; SCI: spinal cord injury.

**Rise of spinal cord organoid models:** Organoid models have become prominent tools to enhance cell diversity in cultures and develop physiologically complex systems for studying cell-to-cell interactions in clinical neuroscience. They also enable longer maturation timescales, supporting late-stage properties such as gliogenesis, myelination, and more complex electrophysiological activity. Detailed protocols and advanced engineering tools to generate hSCOs have been extensively documented (Zhou et al., 2023). Briefly, guided protocols use small molecule concentration gradients that mimic developmental cues of morphogens secreted during embryonic spinal cord formation (**Figure 2**). Neural induction is guided by dual SMAD inhibition, which simultaneously inhibits transforming growth factor beta and bone morphogenic protein signaling pathways. This establishes a population of neural progenitor cells that can undergo subsequent patterning. Axial identity, crucial for neural fate commitment, is established through HOX gene activation, regulated by fibroblast growth factor signaling, growth differentiation factor 11, and time-dependent retinoic acid exposure. Dorso-ventral patterning is generally induced using sonic hedgehog or sonic hedgehog agonist for ventralization, and bone morphogenic protein 4 and WNT signaling for dorsalization. Additionally, inhibition of the NOTCH signaling pathway facilitates the terminal differentiation of neural progenitors into neurons (Buchner et al., 2023).

Resembling human physiology within single organoids remains a challenge, as guided protocols typically generate region-specific organoids with limited in vivo spatial organization. Rigorous quality control measures must be applied to track variability across experimental lines, batches, and individual organoids, ensuring reproducibility and accuracy in the results. This involves monitoring cell viability throughout the differentiation process, evaluating the morphological characteristics such as the size and shape of organoids, and performing gene expression analysis using methods like qPCR and immunocytochemistry. Functional characterization, especially electrophysiological properties, is also critical for assessing the functional integrity of the organoids (Pașca et al., 2025). Advancements in morphogen multiplexing screening, single-cell sequencing technology, and the development of spinal cord atlases from fetal, and adult human tissue have provided critical information regarding spinal cord cellular diversity. These biological tools, combined with engineering technologies like microfluidic platforms, can facilitate the differentiation of a broader range of spinal cord cell types, improving the biofidelity of hSCO models.

Building on these advancements in hSCO differentiation, injury platforms tailor-made for 3D *in vitro* models offer new avenues for studying SCI mechanisms. Biomechanical injuries can be induced through compression using weight drop or platform acceleration, stretch injury via uniaxial or biaxial stretch, static pressure generated in a high-pressure chamber channel, or transection injuries using scratch models. Alternatively, chemical methods such as oxidative stress induction, glucose and oxygen reduction, serum withdrawal, arsenic-induced neurotoxicity, and glutamate-induced excitotoxicity, provide complementary tools to model environmental components of SCI (Stevens et al., 2024). Although the primary clinical mechanism of SCI in humans is contusion, each injury presents unique characteristics and variability in the extent of injury in the spinal cord, making it challenging to generalize treatment strategies and pathological development. This highlights the importance of selecting models guided by specific research questions and desired outcome measures.

Following *in vitro* injury, functional readouts, including electrophysiological responses measured using multielectrode arrays, patch clamping, and calcium imaging, can provide insights into changes in neuronal activity. Evaluation of axonal and cellular integrity, alongside cell viability measurements, provides structural assessments. Additionally, monitoring molecular markers such as cellular stress indicators, mitochondrial dysfunction, and protein release related to clinical biomarkers further helps to characterize the cellular response to injury. These integrated approaches across various readout categories offer a comprehensive understanding of SCI and its potential therapeutic targets.

Assembloids have emerged to enhance model complexity and physiological relevance. The term assembloid refers to the functional integration of two or more organoids. Multi-region neural assembloids combine region-specific organoids like forebrain assembloids, and cortico-spinal-muscle assembloids (Miura et al., 2022). The latter can be integrated into SCI biomechanical models to study disruptions in sensory, motor, and interneuron circuits. While neural organoids readily form new circuits when combined, they currently lack the mature myelination of axons observed in the adult nervous system. This limitation hinders their utility for studying processes such as axon remyelination and impaired axon regeneration.

Enhancing hSCO cellular diversity may be achieved by multi-lineage assembloids. For example, differentiating neural organoids in parallel with vascular cells has been technically challenging as they come from different germ layers. Strategies such as assembloids, vascular cell integration (e.g., endothelial, stromal, and perivascular cells), and vascular-specific growth factor cues can improve organoid health, structural integrity, and functionality. Similarly, neuro-immune assembloids integrating microglia can facilitate the study of inflammation response post-injury and shed light on inflammation dynamics and immune modulation in SCI (Sabate-Soler et al., 2024). By integrating immune, vascular and region-specific neuronal organoids into biomechanical SCI models, deeper understanding of subacute injury phase phenomena, including blood-spinal cord barrier disruption, activation of the inflammatory response and disruption to spinal circuitry can be achieved.

Apart from assembloid biological designs, engineering tools such as 3D printing, electrospinning technologies, and microfluidic devices, can provide both chemical and mechanical cues that influence the structural organization of organoids, enhancing vascular and circuit complexity. For structural support, biomaterials that simulate extracellular matrix environments and vascular-like structures, such as natural (Matrigel) and synthetic polymers with recombinant proteins, are essential.

While these design tools facilitate organoid health and long-term viability, improving organoid maturity remains a biological and engineering challenge. Current hSCO models, at over a month, show neuronal connectivity, enhanced neuron maturation, and glial cells. This is currently the minimum timeframe to introduce *in vitro* injury models, balancing maturation with experimental feasibility. Unlike the postnatal central nervous system in humans, current hSCO models create environments that are highly favorable for axon growth and synaptogenesis. This likely reflects their relatively immature state, including the absence of myelin inhibitors to axon growth since myelination of axons by oligodendrocytes is a process that continues into postnatal development in humans. Notably, human stem cell-based *in vitro* models generally resemble the developmental stage of fetal tissue, which may limit their ability to fully replicate the complexities of mature spinal cord physiology. This developmental immaturity is consistent with findings that disruptions in motor neuron maturation and aging pathways can be identified within gene co-expression networks (Ho et al., 2016). To address this, xenotransplantation of human organoids into animals has shown potential in facilitating neuronal maturation, organoid vascularization, and development of more complex synaptic and intrinsic membrane properties (Revah et al., 2022).

**Conclusion:** Human SCOs provide a transformative platform for studying gray matter pathophysiology and LMN loss after SCI. These *in vitro* human models complement, though cannot fully replace, traditional animal studies by replicating critical aspects of spinal cord function, physiology, and injury response. By enabling detailed exploration of LMN pathology, hSCOs offer valuable insights into neurotrauma mechanisms, motor neuron degeneration, and therapeutic targets. Despite challenges such as immaturity, vascularization, and limited tissue complexity, advancements in bioengineering and single-cell technologies continue to enhance their translational relevance. Leveraging spinal cord organoids for SCI research holds strong potential to advance personalized medicine and neuro-restorative therapies.

*This work was supported by the Belle Carnell Regenerative Neurorehabilitation Fund and by the National Institutes of Health (R01NS113935 to CKF)*.
